# Inhibition of RIPK1-dependent regulated acinar cell necrosis provides protection against acute pancreatitis via the RIPK1/NF-κB/AQP8 pathway

**DOI:** 10.1038/s12276-019-0278-3

**Published:** 2019-08-02

**Authors:** Peng-yu Duan, Yuan Ma, Xi-na Li, Feng-zhi Qu, Liang Ji, Xiao-yu Guo, Wang-jun Zhang, Fan Xiao, Le Li, Ji-sheng Hu, Bei Sun, Gang Wang

**Affiliations:** 1 Department of Pancreatic and Biliary Surgery, The First Affiliated Hospital of Harbin Medical University, Harbin, Heilongjiang Province China; 2 Department of Medical Administration, The First Affiliated Hospital of Harbin Medical University, Harbin, Heilongjiang Province China; 3 Department of Pharmacy, The First Affiliated Hospital of Harbin Medical University, Harbin, Heilongjiang Province China

**Keywords:** Cell death, Cell signalling

## Abstract

Currently, preliminary results have confirmed the existence of receptor-interacting protein kinase 3 (RIPK3) and mixed lineage kinase domain-like protein (MLKL)-dependent necroptosis of pancreatic acinar cells during early acute pancreatitis (AP), which might be a potential target for the effective regulation of necroinflammatory injury. However, the exact effect of receptor-interacting protein kinase 1 (RIPK1)-dependent regulated acinar cell necrosis on AP is still uncertain. In our study, we first explored the changes in the degree of local and systemic inflammation in AP rats when the activation of acinar cell RIPK1 was inhibited. The RIPK1 inhibitor Nec-1 was used to treat rats, and the levels of related inflammatory markers, necrosis indicators and apoptotic indicators were measured. Changes in pancreatic nuclear factor κB (NF-κB) and aquaporin 8 (AQP8) expression were noted. Next, the expression of AQP8 in AR42J cells was inhibited, and the degree of cell necrosis and inflammatory damage was found to be significantly reduced. Most importantly, we demonstrated that the RIPK1/NF-ĸB/AQP8 axis might be a potential regulatory pathway mediating RIPK1-dependent regulated acinar cell necrosis in early AP. Finally, we used the NF-κB inhibitor PDTC and Nec-1 to treat rats in different groups and measured the degree of pathological pancreatic injury, the activation of RIPK1, and the expression of NF-κB and AQP8. In summary, we hypothesized that there might be a RIPK1/NF-ĸB/AQP8 pathway controlling RIPK1-dependent regulated necrosis of acinar cells in AP, which might be a promising therapeutic target against AP-related injury.

## Pancreatitis: Signals that could be silenced

Research into the involvement of a protein and its associated molecular signaling, pathway in a process of cell death called regulated necrosis indicates a possible new approach to treating acute pancreatitis, a life-threatening condition. Gang Wang and colleagues at the First Affiliated Hospital of Harbin Medical University, in China investigated the activity of the protein RIPK1 in pancreatitis in rats. Inhibiting RIPK1 activity in the pancreatic cells that produce digestive enzymes significantly reduced the necrosis and associated damage found in pancreatitis. Inhibiting the production of other proteins which RIPK1 interacts with in a signaling pathway had similar effects. Understanding the detailed mechanisms behind the findings requires further research. The results suggest, however, that drugs controlling RIPK1 activity and its associated signaling pathway may be useful for treating pancreatitis and preventing it from becoming life-threatening.

## Introduction

Acute pancreatitis (AP) is a dangerous and lethal acute abdominal disease with high mortality. AP often progresses rapidly from a mild, self-limited disease to a severe, life-threatening disease with an unfavorable prognosis^1,2^. During the early stage of AP, the death modality of pancreatic acinar cells and inflammatory lesions are the key factors that determine the disease’s course and prognosis^3^. Necrosis and apoptosis are the two major death modes of pancreatic acinar cells in AP. Many studies have shown that the severity of AP is negatively correlated with acinar cell apoptosis and positively correlated with acinar cell necrosis^4^. Compared with mild AP (MAP), severe AP (SAP) is usually accompanied by the necrosis of a large amount of acinar cells, which in turn leads to intense local necroinflammation in the pancreas and might further induce systemic inflammatory response syndrome (SIRS) and multiple organ dysfunction syndrome (MODS), ultimately significantly augmenting the mortality of the disease^4^. Therefore, effective modulation of acinar cell necrosis during early AP might be a potentially effective measure to suppress the malignant development of AP, which is also an important factor that determines its treatment outcomes.

Necrosis has long been considered a rapid, unregulated, irreversible, and passive cell death process caused by pathologically stimulated cells. As the signal transduction pathway or target of necrosis has not been identified, the mechanisms of necrosis remain uncertain, and effective interventions are difficult. Therefore, necrosis has been considered to have no important research significance in disease treatment and has not received adequate attention. In recent years, the traditional concept of necrosis has been critically challenged. A programmed form of cell necrosis, regulated necrosis, has become an important research topic in the fields of inflammation and immune diseases and resulting in a breakthrough in the knowledge and understanding about cell death^5–20^. Regulated necrosis has characteristics common to both necrosis and apoptosis, i.e., it is actively regulated by multiple genes and is accomplished in an orderly and regular manner through the activation of a specific death signaling pathway. In addition, regulated necrosis has the morphological features, subcellular changes and lack of metabolic functions typical of cell necrosis^16^. Therefore, the discovery of necroptosis might provide a potential target for effectively controlling inflammatory lesions and improving disease outcomes for typical necrosis-related diseases.

Regulated necrosis is usually composed of necroptosis, pyroptosis, parthanatos, ferroptosis, and other programs. Among these programs, necroptosis has become a popular topic, attracting increasing attention worldwide. Necroptosis is a kind of regulated necrosis dependent on receptor-interacting protein kinase 3 (RIPK3) and mixed lineage kinase domain-like protein (MLKL)^7^. There is an absolute or relative lack of caspase-8 involvement, and RIPK1 commonly binds to RIPK3 to form the necrosome and initiate a caspase-independent necroptosis process^21,22^. When the necrosome is further phosphorylated, the signal is further transferred downstream to activate MLKL in the cytoplasm, thereby ultimately resulting in necroptosis^23^. Thus, the RIPK1/RIPK3/MLKL pathway should be a key pathway of cellular necroptosis regulation. To date, necroptosis has been observed in pancreatic acinar cells during early AP, and it might be a potential target for the effective inhibition of acinar cell necrosis and noticeable improvement of histological pancreatic injury^24–29^. However, the interventional targets are focused only on RIPK3 and MLKL. The effect of RIPK1-dependent regulated necrosis of acinar cells on AP is still not fully clarified. Thus, we designed the present study to investigate the effect of inhibiting the activation of RIPK1 in pancreatic acinar cells on the early progression of AP both in vivo and in vitro. In addition, we aimed to explore the potential mechanism involved in this process.

## Materials and methods

### Cell cultures

The rat pancreatic exocrine cell line AR42J was purchased from the American Type Culture Collection (Manassas, VA, USA) and cultured in Dulbecco’s modified Eagle’s medium (Gibco, Grand Island, NY, USA) supplemented with 10% fetal bovine serum (ScienCell, San Diego, CA, USA), 100 U/ml penicillin and 100 mg/ml streptomycin (Invitrogen, Carlsbad, CA, USA) at 37 °C in a 5% CO_2_ humidified incubator.

### Reagents

Necrostatin-1 (Nec-1, a selective inhibitor of RIPK1), pyrrolidine dithiocarbamate (PDTC, an inhibitor of NF-κB), cerulein, sodium taurocholate (Na-TC), and sodium pentobarbital were purchased from Sigma-Aldrich (St. Louis, MO, USA). Synthesized duplex siRNAs against AQP8 were purchased from RiboBio Co. Ltd. (Guangzhou, China). Double-stranded siRNA was designed (sense, 5′-GGCCAGUAUUGAGAUUGAUTT-3′; antisense, 5′-AUCAAUCUCAAUACUG GCCTT-3′) with two thymidine residues introduced at the 3′ end. A nonspecific control siRNA (sense, 5′-UUCUCCGAACGUGUCACGU-3′; antisense 5′-ACGU GACACGUUCGGAGAA -3′) was also produced.

### Model establishment and ethics statement

One hundred and twenty male Wistar rats weighing 200–250 g were supplied by the Animal Research Center at the First Affiliated Hospital of Harbin Medical University (Harbin, China). A rat model of AP was established using a previously described method^4,30–34^. Briefly, rats were anesthetized by intraperitoneal injection of sodium pentobarbital (40 mg/kg). AP was induced by a retrograde infusion of 3.5% sodium taurocholate (Na-TC, 0.15 ml/100 g) into the pancreaticobiliary duct. All animal care and experimental protocols were approved by the Institutional Animal Care and Use Committee of Harbin Medical University and were conducted in accordance with the Guide for the Care and Use of Laboratory Animals.

### In vivo experimental design

The rats were randomly allocated into four groups: the AP group (*N* = 15, subjected to the abovementioned procedure), AP + Nec-1 group [*N* = 15, administered an intravenous injection of a Nec-1 solution (1%, 3 mg/kg) via the penile dorsal vein 1 h after AP induction], AP + DMSO group [*N* = 15, administered an intravenous injection of a DMSO solution (0.3 ml/kg) via the penile dorsal vein 1 h after AP induction] and sham group (*N* = 15, subjected to only a midline laparotomy and separation of pancreaticobiliary duct). In the next part of the in vivo experiment, 60 male Wistar rats were randomly allocated into the following five groups, with an average of 15 rats per group: the sham group, AP group, AP + Nec-1 group, AP + PDTC group, and AP + PDTC + Nec-1 group. Rats in the AP + PDTC group was administered PDTC solution (25 mg/kg) via the penile dorsal vein 30 min before the AP model was established. Rats in the AP + PDTC + Nec-1 group was administered PDTC solution via the penile dorsal vein 30 min before the AP model was established, and Nec-1 was administered to the rats via intravenous injection (1%, 3 mg/kg) into the penile dorsal vein after the AP model was established. The doses were selected on the basis of previous reports^35,36^ and our preliminary experiments. In the third part of the in vivo experiment, 60 male rats were randomly divided into four groups with an average of 15 males in each group: the sham group, AP group, AP + si-RIPK1 group, and AP + si-NC group. Rats in the AP + si-RIPK1 and AP + si-NC groups were administered rat cholesterol-conjugated RIPK1 siRNA or si-NC, respectively, in vivo (100 nM) by intravenous injection once a day three times before the AP model was established. The RIPK1 siRNA and si-NC for in vivo delivery were obtained from RiboBio Co. (Guangzhou, China). The serum of every rat was obtained after centrifugation at 3000 × *g* for 15 min and was then stored at −80 °C until assayed.

### Measurement of tissue and serum parameters

The level of serum C-reactive protein (CRP) was measured with an automatic biochemical analyzer (Toshiba, Tokyo, Japan) as previously described^32^. Tumor necrosis factor α (TNF-α), interleukin 18 (IL-18), interleukin 1β (IL-1β), interleukin 6 (IL-6) and cell culture supernatant lactate dehydrogenase (LDH), and TNF-α levels were measured via ELISA kits (R&D Systems, Minneapolis, MN, USA) according to the manufacturer’s instructions. The pancreatic levels of MDA, LDH, MPO and LPO were measured using kits (Jian Cheng, Nan Jing, China) according to the manufacturer’s instructions.

### Hematoxylin and eosin (H&E) staining

H&E staining was performed to visualize the level of inflammation and tissue damage under a light microscope (40×). The scoring system defined by Kusshe et al.^37^ was used, and the final scores of each histopathological examination were collected.

### Terminal deoxynucleotidyl transferase dUTP nick end labeling (TUNEL) assay

The TUNEL assay was conducted using a TUNEL detection kit according to the manufacturer’s instructions (HRP kit DBA; Apotag, Milan, Italy). Briefly, sections were incubated with 15 μg/ml proteinase K for 15 min at room temperature. Endogenous peroxidase was inactivated with 3% H_2_O_2_ for 5 min at room temperature. Sections were immersed in terminal deoxynucleotidyl transferase and biotinylated dUTP in TdT buffer and incubated in a humidified atmosphere at 37 °C for 90 min. Sections were incubated at room temperature for 30 min with horseradish peroxidase-conjugated antibodies, and signals were visualized with diaminobenzidine. The number of TUNEL-positive cells per high-power field (400×) was counted in 5–10 fields for each coded slide. AR42J cells were seeded in 24-well plates. Next, different treatments were added to the cells. Then, a One Step TUNEL Apoptosis Assay Kit (Beyotime Biotechnology, Shanghai, China) was used to evaluate apoptosis following the manufacturer’s instructions. The staining intensity was measured by fluorescence microscopy.

### Transmission electron microscopy

Transmission electron microscopy (TEM) was performed as described previously^30^. Fixed samples were dehydrated through a graded series of ethanols and embedded in epoxy resin. Ultrathin sections (80 nm) were collected on copper grids, double-stained with uranyl acetate and lead citrate, and examined under a Hitachi H-7100 transmission electron microscope (Hitachinaka, Japan) at 80 kV.

### Electrophoretic mobility shift assay (EMSA)

Nuclear extracts from pancreatic tissues (100 mg) were prepared using a nuclear extraction kit (Invitrogen, Carlsbad, CA, USA) according to the manufacturer’s instructions. The binding reactions consisted of 12.5 nM HEPES (pH 7.9), 50–100 mM NaCl, 5% glycerol, 2 mg/mL BSA, 2 μg of poly-dIdC, 10 μg of BSA, 0.1 mM EDTA, 0.1 mM DTT, 1 ng of ^32^P-end-labeled double-stranded DNA probe, and 15 μg of nuclear protein. The binding reactions were incubated for 30 min at 21 °C and were then loaded onto 5% acrylamide-0.25 × Tris-borate-EDTA gels and electrophoresed at 200 V for 2 h. EMSA was carried out with consensus probes specific for NF-κB from Santa Cruz Biotechnology.

### Cell model establishment

To establish the AP model in vitro, AR42J cells were treated with 10^−8^ M cerulein based on previous reports and our preliminary experiments^30^. For the control group, cells were treated with an volume of PBS equivalent to that of cerulein in the AP group. The AP + si-AQP8 and AP + si-NC groups were treated with si-AQP8 or si-NC (50 nM), respectively, for 12 h before AP induction. Cell viability was measured with a Cell Counting Kit-8 (Dojindo Molecular Technologies, Kumamoto, Japan), and no significant changes were observed upon the aforementioned treatments.

### Immunofluorescence

AR42J cells from the control group, AP group, AP + si-AQP8 group, and AP + si-NC group were seeded in 24-well plates. Cells were fixed with 4% paraformaldehyde for 30 min and permeabilized with 0.5% Triton X-100 for 20 min. After incubation with antibodies against Caspase-3, Caspase-8, and Caspase-9 (Abcam) for 2 h, the cells were washed with PBS three times. Then, the cells were incubated with secondary antibodies for 1 h (Beyotime Biotechnology, Shanghai, China), and 4'6-diamino-2-phenylindole (DAPI, Beyotime Biotechnology, Shanghai, China) was added to stain cell nuclei. Finally, the cells were examined by laser scanning confocal microscopy (Olympus).

### ATP assays

The ATP content was determined by using an Enhanced ATP Assay Kit (S0027) according to the manufacturer’s instructions (Beyotime Biotechnology, Shanghai, China). The concentration of ATP was calculated according to an ATP standard curve and expressed as nmol/OD_730._

### Flow cytometry

Cell apoptosis was analyzed by an Annexin V assay kit according to the instructions outlined by the manufacturer (B&D). Briefly, cells were harvested with trypsin, washed twice in PBS and counted. Then, 1 × 10^5^ cells were resuspended in binding buffer at a concentration of 1 × 10^6^ cells/mL. Next, 10 μL of Annexin V and 5 μL of PI were added, and the cells were incubated at room temperature for at least 15 min in the dark. After incubation, the percentage of apoptotic cells was analyzed by flow cytometry (Epics Altra II, Beckman Coulter, USA).

### Transient transfection and RNA interference

Transfections were performed according to the manufacturer’s instructions. To knock down AQP8, synthesized duplex siRNAs (si-AQP8 and si-NC, RiboBio) were used. AR42J cells were grown to 50% confluence in six-well plates and transfected with the siRNAs in serum-free medium without antibiotic supplements using X-treme GENE siRNA transfection reagent (RiboBio, Guangzhou, China). Silencing of protein expression was confirmed by subsequent immunoblot analysis. After transfection, cells were treated with cerulein (24 h) under the aforementioned conditions and were then harvested for further experiments as indicated.

### Real-time polymerase chain reaction analysis

Real-time quantitative polymerase chain reaction (PCR) was used to analyze the Caspase-3, Caspase-8, and Caspase-9 mRNA levels in pancreatic tissues, as described previously^38^. Total RNA was extracted from the cell pellet using an RNA Extraction Kit (Invitrogen, Camarillo, CA, USA) and converted to first-strand cDNA according to the manufacturer’s instructions (Toyobo, Shanghai, China). Real-time PCR was performed using a SYBR^®^ Prime-Script^TM^ RT-PCR Kit in a LightCycler System (Roche Diagnostics, Lewes, UK). The primer sequences were designed by Primer 5.0 and purchased from Invitrogen. The following primer sequences were used for PCR: Caspase-3: sense, 5′-TGTTTGTGTGCTTCTGAGCC-3′ and antisense, 5′-CACG CCATGTCATCATCAAC-3′; Caspase-8:sense, 5′-GATCAAGCCCCACGATGAC-3′ and antisense 5′-CCTGTCCATCAGTGCCATAG-3′; Caspase-9: sense, 5′-CAT TTCATGGTGGAGGTGAAG-3′ and antisense, 5′-GGGAACTGCAGGTGG CTG-3′; AQP8: sense, 5′-TCCTGAGGAGAGGTTCTGGA-3′ and antisense 5′-AGG GCCCTTTGTCTTCTCAT-3′; NF-κB p65: sense, 5′-AGCGTGGGGACTACGACCT -3′ and antisense, 5′-GGGGCACGATTGTCAAAGAT-3′; and GAPDH: sense, 5′-TG GAGTCTACTGGCGTCTT-3′ and antisense 5′-TGTCATATTTCTCGTGGTTCA-3. Amplification was performed with the following cycles: 95 °C for 30 s, followed by 40 cycles of denaturing at 95 °C for 5 s and annealing at 60 °C for 20 s. All reactions were performed in triplicate. Data analysis was performed using the 2^−△△CT^ method, and GAPDH was used as the reference gene.

### Western blotting

The Western blot assay has been previously described^4,39^. In brief, pancreatic tissues or cells were homogenized in protein lysis buffer that contained a protease inhibitor (Beyotime Biotechnology, Shanghai, China) and a phosphatase inhibitor (Roche, Shanghai, China), and debris was removed by centrifugation. Samples were resolved on polyacrylamide sodium dodecyl sulfate gels and electrophoretically transferred to polyvinylidene difluoride membranes. Membranes were blocked with 5% skim milk and incubated with primary antibodies against IL-1β, high-mobility group protein B1 (HMGB1, Cell Signaling Technology), Caspase-1, intercellular adhesion molecule 1 (ICAM-1), NF-κB p65 (p65), phospho-NF-κB p65 (p-p65) (Cell Signaling Technology), IL-18 (Santa Cruz), TNF-α, AQP8, RIPK1, RIPK3, MLKL (Abcam), p-RIPK1, p-RIPK3, p-MLKL (Cell Signaling Technology), Caspase-3, Caspase-8, Caspase-9 (Abcam), and horseradish peroxidase-conjugated secondary antibodies (1:2000, ZSGB-BIO). Unless noted otherwise, the dilution and manufacturer of the abovementioned antibodies were 1:1000 and Abcam, respectively. Immunostained bands were detected using enhanced chemiluminescence kits (Pierce Chemical, Rockford, IL, USA). β-Actin (1:2000, Santa Cruz, CA, USA) was used as the protein loading control, and the relative level of protein expression was calibrated to the relative band density of β-actin.

### Statistical analysis

Data are presented as the means ± S.D.s of three independent experiments and were analyzed using SAS 9.1 for Windows (SAS Institute, Cary, NC, USA). The data were analyzed using one-way ANOVA followed by the Scheffe test. A *p*-value of <0.05 was considered statistically significant.

## Results

### Inhibition of RIPK1 attenuated AP-related injury in rats

**Fig. 1:**
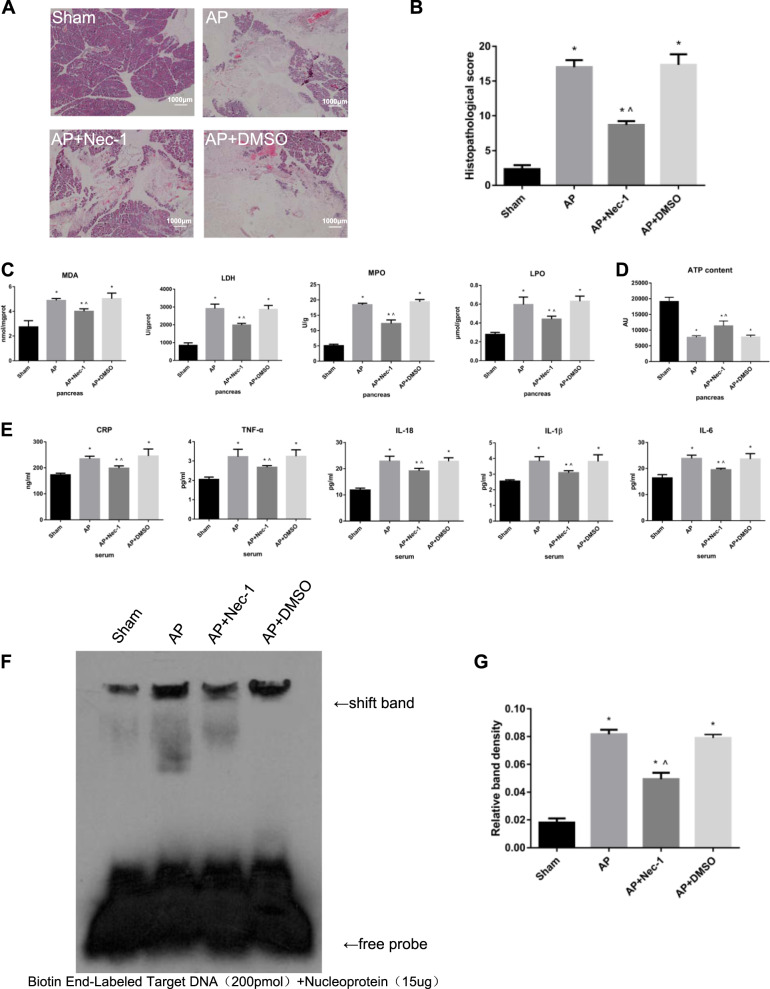
Inhibition of RIPK1 attenuated AP-related injury in rats. **a** Representative images (4×) of hematoxylin and eosin-stained pancreatic tissues harvested from rats subjected to sham operation, AP, AP + Nec-1 or AP + DMSO for 24 h after AP induction. **b** Histopathological scoring was performed to evaluate pancreatic injury in the rats as described above. **c** The levels of MDA, LDH, MPO, and LPO in pancreatic tissues harvested from the rats described above were assessed spectrophotometrically. **d** ATP levels were quantified in pancreatic tissues harvested from the rats. **e** The levels of CRP, TNF-α, IL-18, IL-1β, and IL-6 in peripheral blood samples harvested from the rats described above were spectrophotometrically measured. **f** EMSA analysis and **g** quantitation of NF-κB DNA-binding activity in pancreatic tissues harvested from the rats described in Fig. 1a. The data are presented as the means ± S.Ds. (*n* = 3). *P < 0.05 versus sham, ^*P* < 0.05 versus AP

**Fig. 2:**
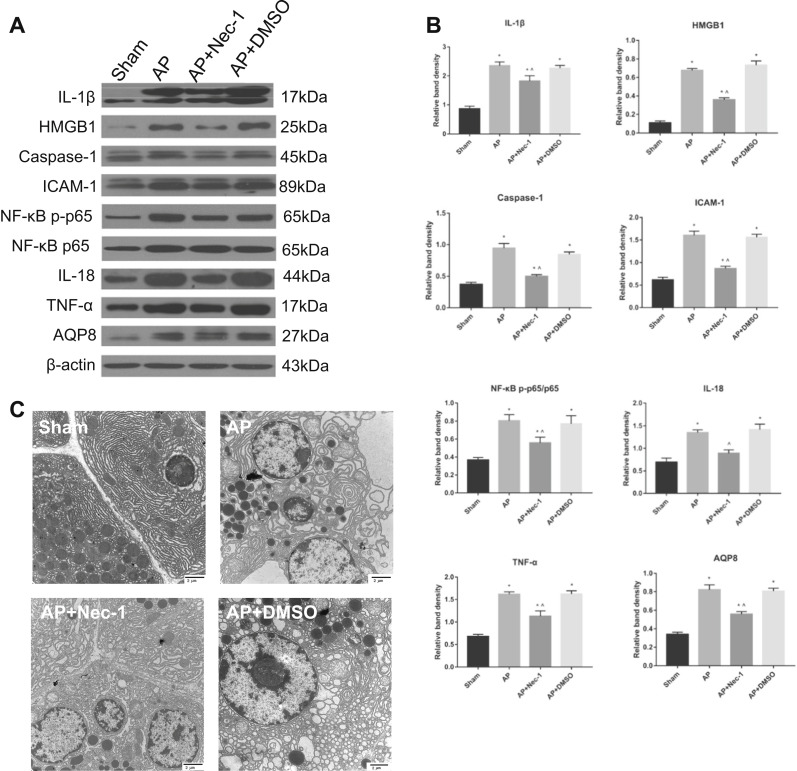
Inhibition of RIPK1 attenuated AP-related injury in rats. **a** Representative immunoblot images and **b** quantitation of IL-1β, HMGB1, Caspase-1, ICAM-1, NF-κB p-p65/p65, IL-18, TNF-α, and AQP8 expression in pancreatic tissues harvested from the rats described above. β-Actin was used as the protein loading control. **c** Representative TEM images of pancreatic tissues from the rats described in Fig. 1a; bar: 2 μm. The data are the means ± S.Ds. (*n* = 3). *P < 0.05 versus sham, ^*P* < 0.05 versus AP

Microscopically, the morphology and structure of the rat pancreatic tissue in the sham group were normal, and no appreciable changes were observed. In the AP group, the tissue showed obvious pancreatic edema, massive hemorrhage, necrosis, indistinct structures of acini and lobules, and a large number of neutrophils. The extent of the pancreatic tissue lesions in the DMSO intervention group was not different from that in the AP group. In the AP + Nec-1 group, the range of necrosis in the pancreas was significantly decreased, the degree of bleeding and edema was reduced, and neutrophil infiltration was slight compared with these parameters in the AP/AP + DMSO group. The pathological scores of pancreatic tissue in the AP, AP + Nec-1, and AP + DMSO groups after 24 h of treatment showed that the pathological changes were the most severe and the pancreatic tissue pathological scores were the highest in the AP and AP+ DMSO groups, while the pathological changes and scores in the Nec-1 intervention group were the mildest and lowest, respectively; in addition, the difference was significant (Fig. 1a, b). Compared with rats in the AP group, rats in the AP + Nec-1 group exhibited significantly decreased expression of MDA, LDH, MPO, and LPO in the pancreas, while rats in the AP + DMSO group showed no difference (Fig. 1c). The ATP content in the pancreas of rats in the four experimental groups was measured. Compared with rats in the sham group, rats in the AP, AP + Nec-1, and AP + DMSO groups had a significantly reduced ATP content in the pancreatic tissue, which demonstrated the degree of cell necrosis. The content of ATP in the pancreas of AP + Nec-1 group rats was significantly increased compared with that in AP group rats, but there was no significant difference between the AP + DMSO group and the AP group (Fig. 1d). We collected serum from the rats in the four groups after 24 h of treatment. The serum levels of CRP, TNF-α, IL-18, IL-1β, and IL-6 in the AP, AP + Nec-1, and AP + DMSO groups were significantly increased compared with those in the sham group. Administration of Nec-1 significantly reduced the serum CRP, TNF-α, IL-18, IL-1β, and IL-6 concentrations compared with those in the AP group, but there was no significant difference between the serum levels of these molecules in the AP + DMSO group and the AP group (Fig. 1e). The NF-κB DNA-binding activity in rat pancreatic tissue was also detected by EMSA and was found to be significantly decreased in the Nec-1 intervention group compared with that in the AP group (Fig. 1f, g). The expression levels of inflammatory mediators (IL-1β, HMGB1, Caspase-1, ICAM-1, NF-κB p-p65, IL-18, and TNF-α) were significantly increased in the AP group compared with those in the other groups. The expression levels of inflammatory mediators in the AP + Nec-1 group were significantly reduced compared with those in the AP group, but did not significantly change in the AP + DMSO group (Fig. 2a, b). The subcellular structure of acinar cells was observed via TEM. In the sham group, the cell membrane of normal acinar cells was smooth and intact, the intercellular space was clear, the endoplasmic reticulum was arranged neatly, and the mitochondrial surface was smooth and round. In the AP group and AP + DMSO group, the structure of the endoplasmic reticulum in pancreatic acinar cells was disordered, and a large amount of endoplasmic reticulum was absent. The surface of mitochondria was wrinkled, and membrane structures from large amounts of degraded cell components were observed. In comparison, the irregularities in the endoplasmic reticulum and mitochondria seen in the AP + Nec-1 group were significantly milder (Fig. 2c). The above experiments indicated that inhibiting RIPK1 with Nec-1 alleviated the extent of pancreatic damage and the systemic inflammatory response and played a protective role in AP in vivo.

**Fig. 3:**
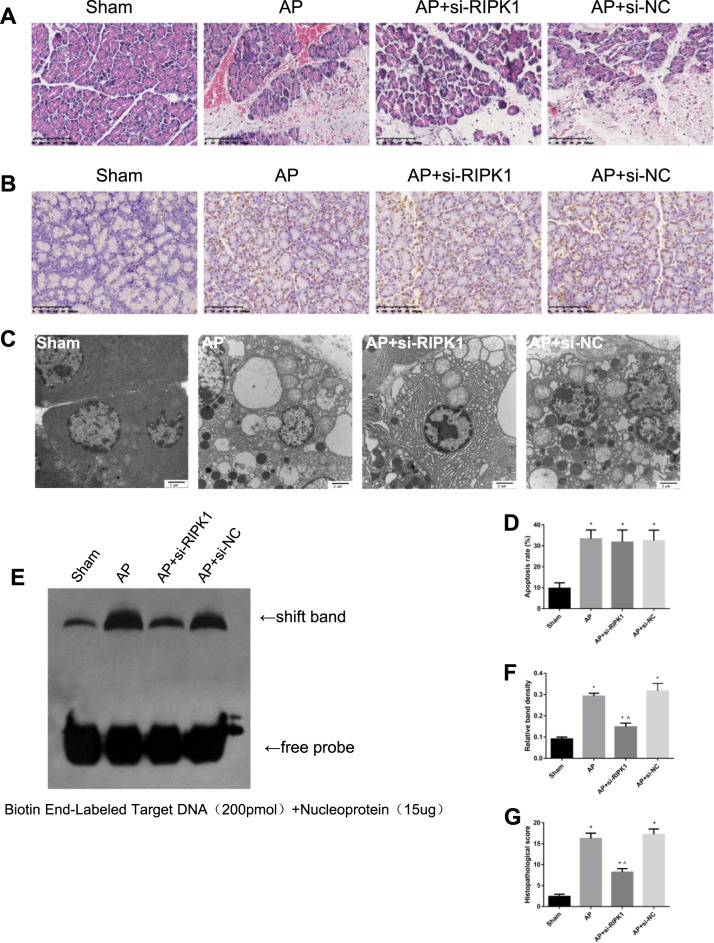
Inhibition of RIPK1 attenuated AP-related injury in rats. **a** Representative images (4×) of hematoxylin and eosin-stained pancreatic tissues harvested from rats subjected to sham operation, AP, AP + si-RIPK1 or AP + si-NC for 24 h after AP induction. **b** TUNEL analysis of apoptotic cells and **d** the apoptosis index in pancreatic tissues harvested from the rats described in Fig. 3a. **c** Representative TEM images of pancreatic tissues from the rats described in Fig. 3a; bar: 2 μm. **e** EMSA analysis and **f** quantitation of NF-κB DNA-binding activity in pancreatic tissues harvested from the rats described in Fig. 3a. **g** Histopathological scoring was performed to evaluate pancreatic injury in the rats described above. The data are the means ± S.Ds. (*n* = 3). *P < 0.05 versus sham, ^*P* < 0.05 versus AP

**Fig. 4:**
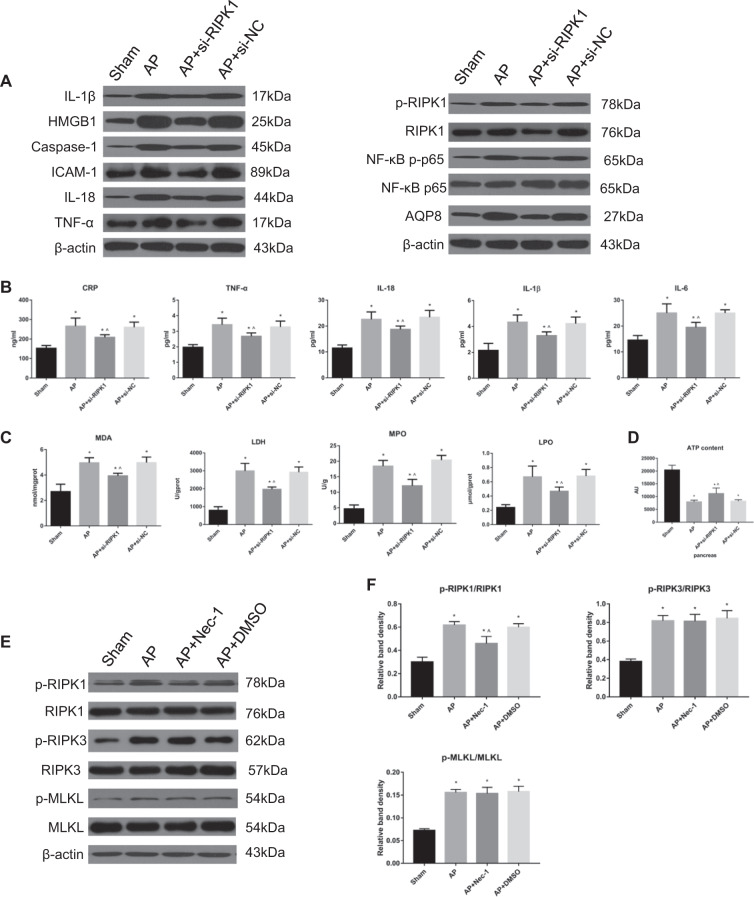
Inhibition of RIPK1 did not affect the classic necroptosis pathway in AP rats. **a** Representative immunoblot images and **b** quantitation of the IL-1β, HMGB1, Caspase-1, ICAM-1, IL-18, TNF-α, p-RIPK1/RIPK1, NF-κB p-p65/p65, and AQP8 levels in pancreatic tissues harvested from the rats described above in Fig. 3a. β-Actin was used as the protein loading control. **b** The levels of CRP, TNF-α, IL-18, IL-1β, and IL-6 in peripheral blood samples harvested from the rats described in Fig. 3a were spectrophotometrically measured. **c** The levels of MDA, LDH, MPO, and LPO in pancreatic tissues harvested from the rats described above in Fig. 3a were assessed spectrophotometrically. **d** The ATP levels in pancreatic tissues harvested from the rats in Fig. 3a were quantified. **e** Representative immunoblot images and **f** quantitation of the p-RIPK1/RIPK1, p-RIPK3/RIPK3, and p-MLKL/MLKL ratios in pancreatic tissues harvested from the rats described in Figure 3a. β-Actin was used as the protein loading control. The data are the means ± S.Ds. (*n* = 3). *P < 0.05 versus sham, ^*P* < 0.05 versus AP

In the third part of the in vivo experiment, different groups of rats were administered rat cholesterol-conjugated RIPK1 siRNA or si-NC in vivo (100 nM) by intravenous injection. In the sham group, AP group, AP + si-RIPK1 group, and AP + si-NC group, we performed H&E staining (Fig. 3a, g); detected the expression of MDA, LDH, MPO, and LPO in the pancreas (Fig. 4c); measured the content of ATP in the pancreas (Fig. 4d); and measured the serum levels of CRP, TNF-α, IL-18, IL-1β, and IL-6 (Fig. 4b). In addition, the NF-κB DNA-binding activity in rat pancreatic tissue was detected by EMSA (Fig. 3e, f), and the expression levels of inflammatory mediators (IL-1β, HMGB1, Caspase-1, ICAM-1, IL-18, TNF-α, and NF-κB p-p65) were measured by Western blotting (Fig. 4a). The subcellular structure of acinar cells was observed via TEM (Fig. 3c). These results showed that the intravenous tail vein injection of cholesterol-conjugated si-RIPK1 and the inhibition of RIPK1 activation by Nec-1 had consistent effects on attenuating the extent of pancreatic damage and systemic inflammatory response in vivo.

### Inhibition of RIPK1 did not affect the classic necroptosis pathway in AP rats

To observe the classic RIPK1/RIPK3/MLKL necroptosis pathway in AP rats, RIPK1, RIPK3, and MLKL phosphorylation was assessed by Western blotting, and we found that the RIPK1 phosphorylation (activation) levels significantly decreased after treatment with Nec-1, while those of RIPK3 and MLKL did not change appreciably (Fig. 4e, f), which meant that the classic necroptosis pathway was not affected.

### Inhibition of RIPK1 during AP did not affect apoptosis in rats

**Fig. 5:**
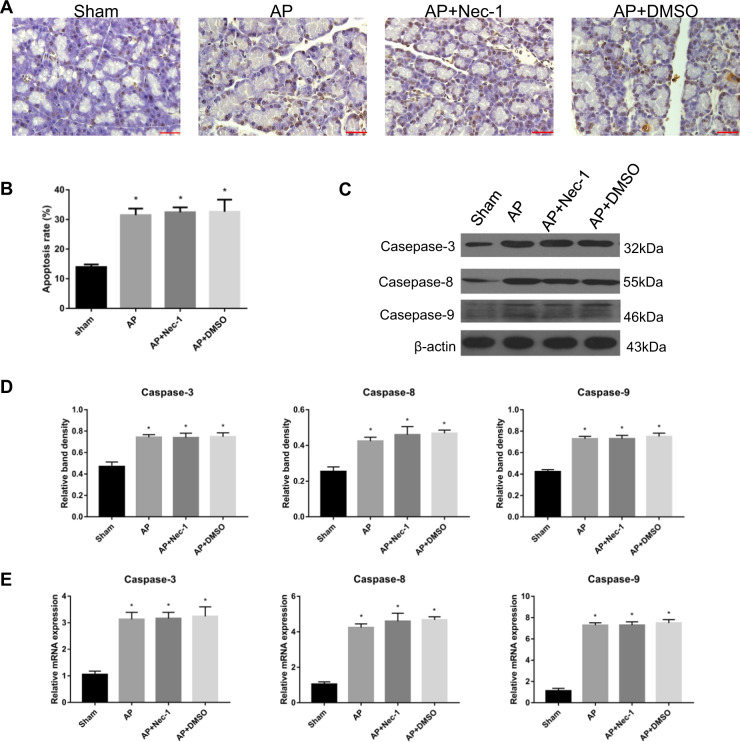
Inhibition of RIPK1 during AP did not affect apoptosis in rats. **a** TUNEL analysis of apoptotic cells and **b** the apoptosis index in pancreatic tissues harvested from the rats described in Fig. 1a. **c** Representative immunoblot images and **d** quantitation of Caspase-3, Caspase-8, and Caspase-9 expression in pancreatic tissues harvested from the rats described in Fig. 1a. β-Actin was used as the protein loading control. **e** Relative mRNA expression of Caspase-3, Caspase-8, and Caspase-9 in pancreatic tissues harvested from the rats described in Fig. 1a. The data are the means ± S.Ds. (*n* = 3). *P < 0.05 versus sham

A TUNEL assay was used to study the apoptosis of pancreatic tissue in each group. We found that there was no significant difference between the AP group and the AP + Nec-1 group (Fig. 5a, b). The protein expression and relative mRNA expression of the apoptosis-related mediators Caspase-3, 8, and 9 showed the same results as those of the TUNEL assay in the abovementioned tissues (Fig. 5c–e). In another group of in vivo experiments, we also performed TUNEL assay. The results showed that there was no significant change in apoptotic degree of AP group, AP + si-RIPK1 group and AP + si-NC group (Fig. 3b, d).Therefore, pancreatic apoptosis was not affected by the inhibition of RIPK1.

### RIPK1 regulated cell necrosis and inflammatory damage in AP through AQP8

**Fig. 6:**
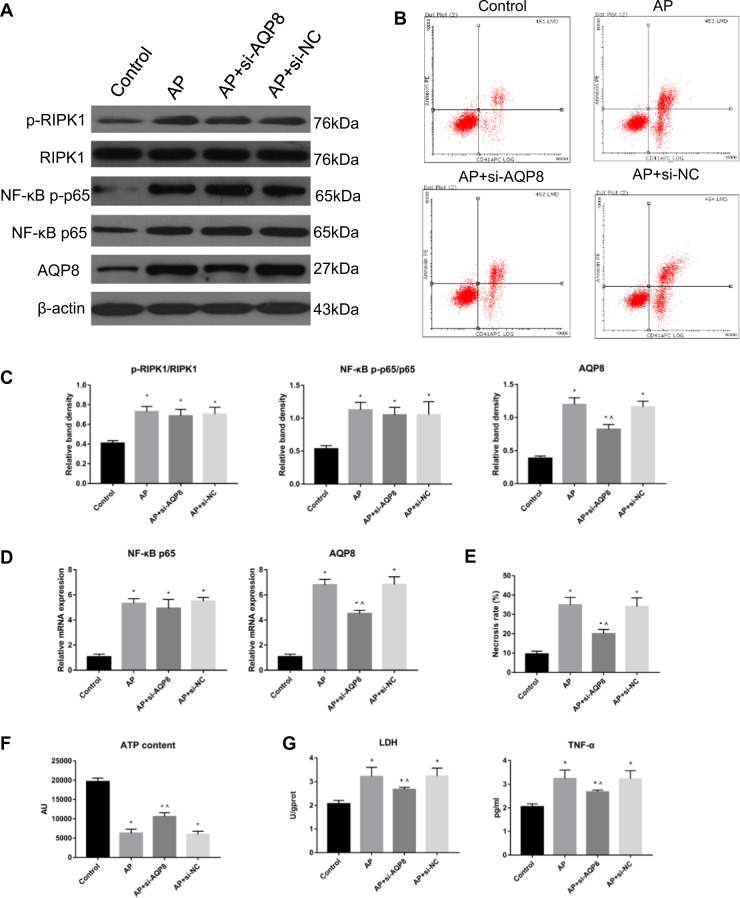
RIPK1 regulated cell necrosis and inflammatory damage in AP through AQP8. **a** Representative immunoblot images and **c** quantitation of the p-RIPK1/RIPK1, NF-κB p-p65/p65, and AQP8 levels in AR42J cells from the Control, AP, AP + si-AQP8 and AP + si-NC groups. β-Actin was used as the protein loading control. **b** Representative flow cytometry results for cell necrosis in the AR42J cells described in Fig. 6a. **d** Relative mRNA expression of NF-κB p65 and AQP8 in the AR42J cells described in Fig. 6a. **e** Histograms showing the necrosis rates of the AR42J cells described in Fig. 6b, as measured by flow cytometry. **f** ATP levels were quantified in the AR42J cells described in Fig. 6a. **g** The levels of LDH and TNF-α in AR42J cell culture supernatants as described in Fig. 6a were assessed spectrophotometrically. The data are the means ± S.Ds. (*n* = 3). *P < 0.05 versus control, ^*P* < 0.05 versus AP

**Fig. 7:**
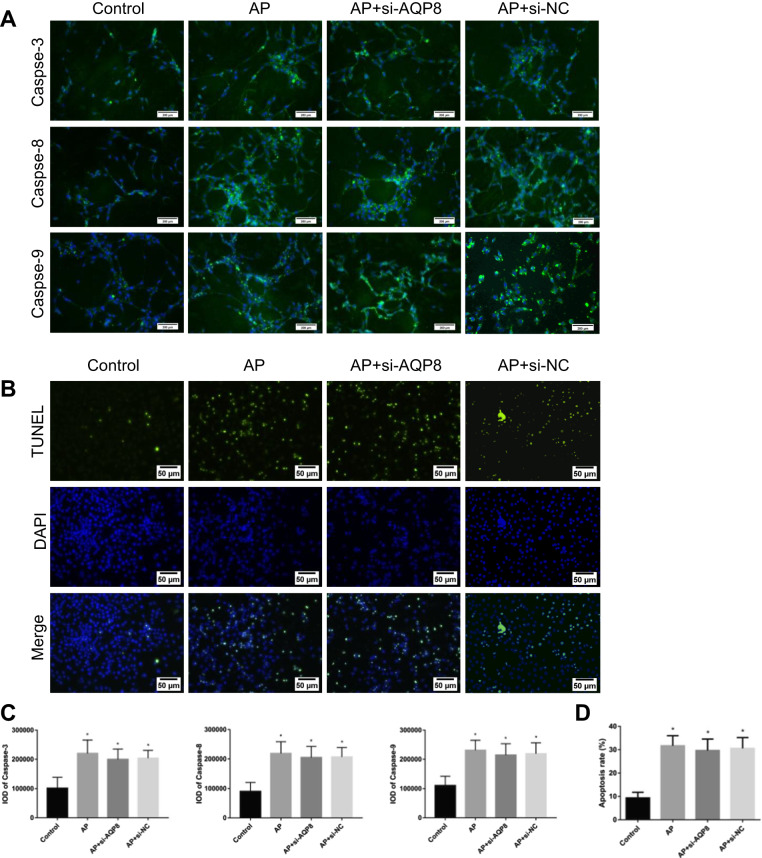
Inhibition of AQP8 during AP did not affect apoptosis in vitro. **a** Immunofluorescence staining revealed the expression of Caspase-3, Caspase-8, and Caspase-9 in the cytoplasm of AR42J cells from the Control, AP, AP + si-AQP8 and AP + si-NC groups. The green signal represents Caspase-3, Caspase-8, and Caspase-9 staining. The blue signal represents nuclear DNA staining by DAPI; bar: 200 μm. **b** A TUNEL assay was used to measure apoptosis in AR42J cells and (**d**) determine the apoptosis index in the AR42J cells described in Fig. 7b. **c** The histograms show the IOD of Caspase-3, Caspase-8, and Caspase-9 in the AR42J cells described in Fig. 7a. The data are the means ± S.Ds. (*n* = 3). *P < 0.05 versus control, ^*P* < 0.05 versus AP

In vitro experiments showed that in the control group of AR42J cells, the protein expression and relative mRNA expression of AQP8 were lower than those in the AP group, AP + si-AQP8 group and AP + si-NC group, which increased to different degrees. Compared with the AP group, the AP + si-AQP8 group exhibited significantly decreased protein expression and relative mRNA expression of AQP8. However, the levels of phospho-RIPK1 and NF-κB p-p65 in the AP group, AP + si-AQP8 group and AP + si-NC group were significantly higher than those in the Control group (Fig. 6a, c, d). The contents of LDH and TNF-α in the AR42J cell culture supernatant of the AP group, AP + si-AQP8 group and AP + si-NC group were significantly increased compared with those in the supernatant from control cells, while the contents of LDH and TNF-α in the AP + si-AQP8 group were significantly decreased compared with those in the AP group (Fig. 6g). Flow cytometry showed that among the control group, AP group, AP + si-AQP8 group and AP + si-NC group, the degree of AR42J cell necrosis in the control group was lower than that in the AP group, AP + si-AQP8 group and AP + si-NC group. The cell necrosis rate in the AP group was higher than that in the AP + si-AQP8 group (Fig. 6b, e). Further study showed that the ATP level in control group AR42J cells was higher than that in AP group, AP + si-AQP8 group and AP + si-NC group AR42J cells and that the ATP level in AP + si-AQP8 group cells was higher than that in AP group AR42J cells (Fig. 6f). In addition, immunofluorescence staining of Caspase-3, -8 and -9 and TUNEL experiments in vitro showed that the levels of apoptosis in the AP group, AP + si-AQP8 group and AP + si-NC group of AR42J cells were not significantly different (Fig. 7).

### A RIPK1/NF-κB p65/AQP8 axis might exist during AP in rats

**Fig. 8:**
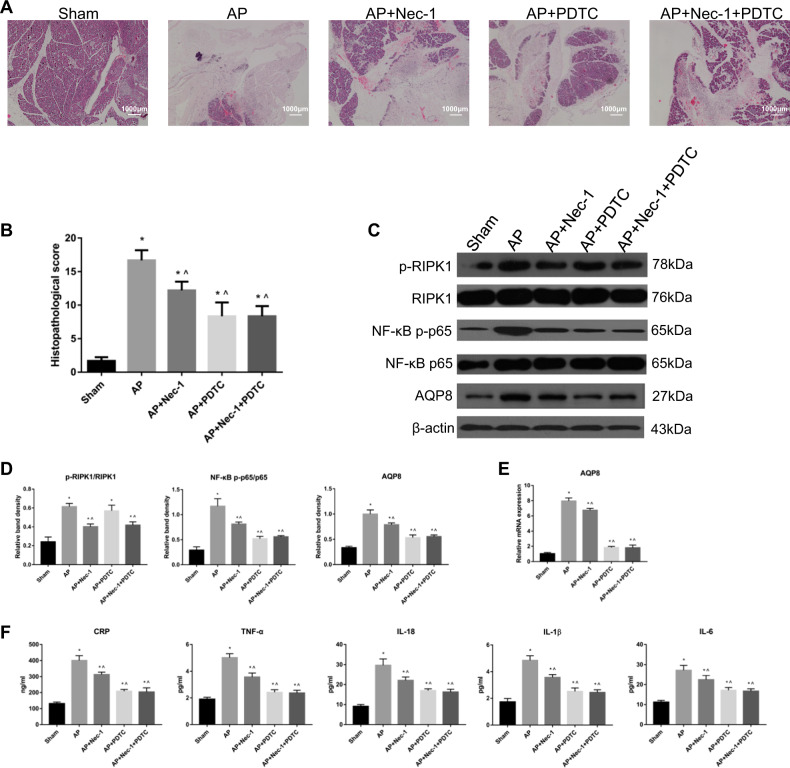
A RIPK1/NF-κB p65/AQP8 axis might exist during AP in rats. **a** Representative images (4×) of hematoxylin and eosin-stained pancreatic tissues harvested from rats subjected to sham operation, AP, AP + Nec-1, AP + PDTC or AP + Nec-1 + PDTC for 24 h after AP induction. **b** Histopathological scoring was performed to evaluate pancreatic injury in the rats described above. **c** Representative immunoblot images and **d** quantitation of the p-RIPK1/RIPK1, NF-κB p-p65/p65, and AQP8 levels in pancreatic tissues harvested from the rats described in Fig. 8a. β-Actin was used as the protein loading control. **e** Relative mRNA expression of AQP8 in pancreatic tissues harvested from the rats described in Fig. 8a. **f** The levels of CRP, TNF-α, IL-18, IL-1β, and IL-6 in peripheral blood samples harvested from the rats described in Fig. 8a were spectrophotometrically measured. The data are the means ± S.Ds. (*n* = 3). *P < 0.05 versus sham, ^*P* < 0.05 versus AP

We found that in the sham, AP, and AP + Nec-1 groups, the levels of NF-κB p-p65 and AQP8 was consistent with the changes in the RIPK1 phosphorylation level in the three groups (Fig. 2a, b, e, f). The activation level of NF-κB p65 in rat pancreatic tissue was also assessed by EMSA and was significantly decreased in the Nec-1 intervention group compared with that in the AP group (Fig. 1f, g). To determine the role of NF-κB p65 activation in RIPK1-mediated necrosis progression in vivo, we established five experimental groups on the basis of the original groups: sham, AP, AP + Nec-1, AP + PDTC, and AP + Nec-1 + PDTC. Microscopically, the morphology and structure of the pancreatic tissue in sham group rats were normal, and no obvious change was observed. In the AP group, pancreatic edema was obvious, along with hemorrhage and necrosis in large areas, unclear structure of acini and lobules and a large number of infiltrated neutrophils. In the AP + Nec-1 group, AP + Nec-1 + PDTC group, and AP + PDTC group, pancreatic necrosis was reduced, bleeding and edema were ameliorated, and only slight infiltration of neutrophils was observed. At 24 h, the pancreatic tissue pathological score showed that in the 5 experimental groups, the pathological changes in the pancreas were the most severe and the pathological score was the highest in the AP group. Although the pathological changes and scores in the AP + Nec-1 group, AP + Nec-1 + PDTC group, and AP + PDTC group were decreased, the changes and scores in the AP + PDTC group were less severe and lower, respectively, than those in the AP + Nec-1 group, and there was no significant difference between the AP + Nec-1 + PDTC group and AP + PDTC group (Fig. 8a, b). The serum CRP, TNF-α, IL-18, IL-1β, and IL-6 concentrations in the five experimental groups were measured by ELISA, and the results were consistent with the trend and degree of pathological damage shown by H&E staining (Fig. 8f). We measured the levels of phospho-RIPK1 and NF-κB p-p65 and the protein and relative mRNA expression of AQP8 in the pancreatic tissue of rats. Compared with these parameters in the AP group, the phosphorylation of RIPK1 in the AP + PDTC group was not significantly different but the expression of AQP8 was significantly decreased. The expression level of AQP8 in the AP + PDTC group and AP + Nec-1 + PDTC group was less than that in the AP + Nec-1 group, and there was no significant difference in the AQP8 level between the AP + PDTC group and AP + Nec-1 + PDTC group (Fig. 8c–e). Therefore, we inferred the presence of a RIPK1/NF-κB p65/AQP8 axis in the pancreas of rats during AP.

## Discussion

The predominant pathophysiological changes of early AP occur in pancreatic acinar cells^40^. MAP and SAP share some common characteristics in acinar cell pathology; however, these two conditions exhibit very different degrees of severity and prognoses. Hence, the secondary response of pancreatic acinar cells to early intracellular damage, namely, the mode of acinar cell death, becomes a critical factor that determines the degree of the inflammatory response as well as the occurrence and development of subsequent complications in AP. Kaiser et al.^41^ showed that pancreatic cells are primarily apoptotic in MAP, with mild inflammation, but predominantly necrotic in SAP, with a severe inflammatory response. In patients with pancreatitis causing detectable necrosis in ≥50% of the pancreas, the mortality rate approaches 20%^42^. Therefore, the effective inhibition of pancreatic acinar cell necrosis during early AP could noticeably suppress the local inflammatory response in the pancreas at its origin and prevent its continuous amplification to limit disease progression.

During AP, routine interventions mainly concentrate on resolving the relevant simultaneous injuries after cell necrosis instead of on directly modulating necrosis, since pancreatic acinar cell necrosis is an acute, unregulated death process and effective intervention is difficult. Necrosis in acinar cells is rapid, concurrent and occurs in clusters during AP^30^. The direct inhibition of acinar necrosis in early AP—specifically, the timely, effective and ultimate suppression of local and systemic inflammation at its origin—may prevent interference from many downstream factors after acinar necrosis occurs. This regulation may reduce the uncertainty and complexity of interventions, and its action would have an immediate effect and induce a rapid onset of biological functions. Necroptosis is a newly discovered cell-regulated necrosis that has been suggested to play a critical role in many pathophysiological conditions, including immune diseases, viral infections, malignant tumors and ischemia-reperfusion^13,16,43^. However, the necroptosis program does not exist in all tissues and cells of living organisms^44^. Currently, some preliminary results have confirmed the existence of pancreatic acinar cell necroptosis during early AP, which might be a potential target for the effective regulation of necroinflammatory injuries. He et al.^24,25,29^ demonstrated that in AP models of RIPK3 and MLKL gene knockout mice, the quantity of necrotic acinar cells, the histological changes in the pancreas and the severity of AP were appreciably reduced. Ma et al.^27^ demonstrated that miR-21 was overexpressed in AP and that its effective inhibition could significantly reduce the severity of AP by inhibiting the RIPK3-dependent necroptosis pathway.

In this study, we used RIPK1 as a potential target and observed its effect on histological changes in the pancreas and inflammatory lesions in AP. The in vivo results showed that Nec-1 and cholesterol-conjugated si-RIPK1 significantly reduced the extent of pancreatic tissue necrosis, inhibited the massive release of inflammatory mediators and oxidative stress damage, and ultimately effectively decreased the severity of AP. Although the regulation of RIPK1 activation in acinar cells had an obvious impact on pancreatic necrosis and the course of AP, it did not have a significant effect on the activation of RIPK3 and MLKL in acinar cells. These results indicated that RIPK1-dependent regulated necrosis of acinar cells is controlled by targets other than the classical RIPK1/RIPK3/MLKL pathway. Linkermann et al.^26^ found that in a mouse model of AP, Nec-1 appreciably enhanced pancreatic damage, which was contradictory to our results. We believe that multiple factors might contribute to the initiation and execution of cell-regulated necrosis, which is a dynamic chain reaction process. The molecular mechanisms by which regulated necrosis is initiated and regulated vary across tissues and cells. Different experimental animals, materials, designs, conditions, methods, and so on might have certain effects on the experimental results.

In early AP, the modalities of acinar cell death mainly include necrosis and apoptosis. Apoptosis is also a programmed form of cell death that can be regulated by extrinsic and intrinsic pathways. In AP, necrosis and apoptosis are not in complete opposition but are in a dynamic and interactive relationship under different interventions and stimuli. Our previous results proved that inhibition of hydrogen sulfide synthesis provided protection for SAP rats via the simultaneous induction of apoptosis and depression of necrosis in acinar cells^45^. In this study, we also investigated whether RIPK1-dependent regulated necrosis of acinar cells had an effect on the apoptotic process in these cells. The results showed that the regulation of RIPK1 levels in acinar cells did not markedly affect either the expression of the apoptosis-related proteins Caspase-3, -8, and -9 or acinar cell apoptosis during AP. The reduction in the degree of pancreatic tissue necrosis accompanied by RIPK1 activation in acinar cells is not achieved through the pathway of acinar cell apoptosis but is realized by the direct and targeted regulation of pancreatic RIPK1-dependent regulated necrosis.

RIPK1 is a member of the RIPK family, and its N terminus contains a serine/threonine protein kinase domain. RIPK1 is also a necessary protein for sensing the internal and external stress signals on various membranes and is an important inflammatory mediator within cells. RIPK1 can regulate various inflammatory targets in the body, such as NF-κB, Akt, AQP8, JNK, and TNF-α^5,15,46^. Aquaporins (AQPs) are a family of channels that used to be known as facilitators of water transport across cell membranes in response to osmotic gradients^47^. Aquaporins selectively transfer water molecules in and out of the cell while preventing the passage of ions and other solutes. Also known as water channels, aquaporins are actually integral membrane pore proteins. Some researchers have assumed that AQPs are tightly involved in inflammatory cytokine release^48,49^. Experimental results showed that AQPs are closely related to the degree of inflammation in AP^50,51^. Ma and Matsuzaki et al.^52,53^ demonstrated the presence of AQP8 in pancreatic acinar cells. In this study, we confirmed that the level of AQP8 in acinar cells was markedly increased in vitro during AP. Inhibition of AQP8 could significantly reduce the degree of necrosis, showing that AQP8 might be a critical target directly inducing acinar cell necrosis. Furthermore, we found that the inhibition of RIPK1 expression in pancreatic acinar cells led to a concomitant reduction in NF-κB activity and AQP8 levels. NF-κB is an important transcription factor in the development and progression of AP and is involved in the modulation of cell death and inflammatory injury in the course of AP. It has been reported that AQP expression might be mediated by the increased activation of NF-κB^54^. Therefore, we hypothesized that the RIPK1/NF-κB/AQP8 axis might be a potential regulatory pathway in RIPK1-dependent regulated necrosis of acinar cells in AP. Therefore, we used Nec-1 and an NF-κB inhibitor (PDTC) to interfere with AP in vivo. We found that the expression trend of AQP8 in acinar cells was consistent with that of NF-κB and RIPK1, indicating that the RIPK1/NF-κB/AQP8 axis might be pathway regulating the occurrence of RIPK1-dependent regulated necrosis. However, the exact molecular mechanism involved in this process remains to be explained more thoroughly in the future.

In summary, RIPK1-dependent regulated necrosis of acinar cells plays a critical role in the early progression of AP. Inhibition of RIPK1 expression in acinar cells effectively alleviates the degree of inflammatory damage and pancreatic histological changes in AP by significantly suppressing RIPK1-dependent regulated necrosis without inducing the apoptosis pathway in acinar cells. This process might be potentially regulated by the RIPK1/NF-κB/AQP8 pathway. However, the definite molecular mechanism needs future investigation.

## References

[1] Wang, G. et al. From nitric oxide to hyperbaric oxygen: invisible and subtle but nonnegligible gaseous signaling molecules in acute pancreatitis. *Pancreas* **43**, 511–517 (2014). 24713669 10.1097/MPA.0000000000000062

[2] Wang, G., Qu, F. Z., Li, L., Lv, J. C. & Sun, B. Necroptosis: a potential, promising target and switch in acute pancreatitis. *Apoptosis* **21**, 121–129 (2016). 26514558 10.1007/s10495-015-1192-3

[3] Kang, R., Lotze, M. T., Zeh, H. J., Billiar, T. R. & Tang, D. Cell death and DAMPs in acute pancreatitis. *Mol. Med.* **20**, 466–477 (2014). 25105302 10.2119/molmed.2014.00117 PMC4277549

[4] Wang, G. et al. Inhibition of hydrogen sulfide synthesis provides protection for severe acute pancreatitis rats via apoptosis pathway. *Apoptosis* **18**, 28–42 (2013). 23054084 10.1007/s10495-012-0770-x

[5] Dannappel, M. et al. RIPK1 maintains epithelial homeostasis by inhibiting apoptosis and necroptosis. *Nature* **513**, 90–94 (2014). 25132550 10.1038/nature13608 PMC4206266

[6] Newton, K. et al. Is SIRT2 required for necroptosis? *Nature* **506**, E4–E6 (2014). 24572428 10.1038/nature13024 PMC4005920

[7] Pasparakis, M. & Vandenabeele, P. Necroptosis and its role in inflammation. *Nature* **517**, 311–320 (2015). 25592536 10.1038/nature14191

[8] Li, S. et al. Pathogen blocks host death receptor signalling by arginine GlcNAcylation of death domains. *Nature* **501**, 242–246 (2013). 23955153 10.1038/nature12436

[9] Takahashi, N. et al. RIPK1 ensures intestinal homeostasis by protecting the epithelium against apoptosis. *Nature* **513**, 95–99 (2014). 25186904 10.1038/nature13706

[10] Newton, K. et al. Activity of protein kinase RIPK3 determines whether cells die by necroptosis or apoptosis. *Science* **343**, 1357–1360 (2014). 24557836 10.1126/science.1249361

[11] Zhang, D. W. et al. RIP3, an energy metabolism regulator that switches TNF-induced cell death from apoptosis to necrosis. *Science* **325**, 332–336 (2009). 19498109 10.1126/science.1172308

[12] Rickard, J. A. et al. RIPK1 regulates RIPK3-MLKL-driven systemic inflammation and emergency hematopoiesis. *Cell* **157**, 1175–1188 (2014). 24813849 10.1016/j.cell.2014.04.019

[13] Zhou, W. & Yuan, J. SnapShot: Necroptosis. *Cell* **158**, 464–464 e461 (2014). 25036639 10.1016/j.cell.2014.06.041

[14] Wang, Z., Jiang, H., Chen, S., Du, F. & Wang, X. The mitochondrial phosphatase PGAM5 functions at the convergence point of multiple necrotic death pathways. *Cell* **148**, 228–243 (2012). 22265414 10.1016/j.cell.2011.11.030

[15] Dillon, C. P. et al. RIPK1 blocks early postnatal lethality mediated by caspase-8 and RIPK3. *Cell* **157**, 1189–1202 (2014). 24813850 10.1016/j.cell.2014.04.018 PMC4068710

[16] Linkermann, A. & Green, D. R. Necroptosis. *N. Engl. J. Med.* **370**, 455–465 (2014). 24476434 10.1056/NEJMra1310050 PMC4035222

[17] Kaczmarek, A., Vandenabeele, P. & Krysko, D. V. Necroptosis: the release of damage-associated molecular patterns and its physiological relevance. *Immunity* **38**, 209–223 (2013). 23438821 10.1016/j.immuni.2013.02.003

[18] Duprez, L. et al. RIP kinase-dependent necrosis drives lethal systemic inflammatory response syndrome. *Immunity* **35**, 908–918 (2011). 22195746 10.1016/j.immuni.2011.09.020

[19] Moriwaki, K. et al. The necroptosis adaptor RIPK3 promotes injury-induced cytokine expression and tissue repair. *Immunity* **41**, 567–578 (2014). 25367573 10.1016/j.immuni.2014.09.016 PMC4220270

[20] Bleriot, C. et al. Liver-resident macrophage necroptosis orchestrates type 1 microbicidal inflammation and type-2-mediated tissue repair during bacterial infection. *Immunity* **42**, 145–158 (2015). 25577440 10.1016/j.immuni.2014.12.020

[21] Strilic, B. et al. Tumour-cell-induced endothelial cell necroptosis via death receptor 6 promotes metastasis. *Nature* **536**, 215–218 (2016). 27487218 10.1038/nature19076

[22] Zhang, Y. et al. RIP1 autophosphorylation is promoted by mitochondrial ROS and is essential for RIP3 recruitment into necrosome. *Nat. Commun.* **8**, 14329 (2017). 28176780 10.1038/ncomms14329 PMC5309790

[23] Lin, J. et al. RIPK1 counteracts ZBP1-mediated necroptosis to inhibit inflammation. *Nature* **540**, 124–128 (2016). 27819681 10.1038/nature20558 PMC5755685

[24] Wu, J. et al. Mlkl knockout mice demonstrate the indispensable role of Mlkl in necroptosis. *Cell Res.* **23**, 994–1006 (2013). 23835476 10.1038/cr.2013.91 PMC3731568

[25] He, S. et al. Receptor interacting protein kinase-3 determines cellular necrotic response to TNF-alpha. *Cell* **137**, 1100–1111 (2009). 19524512 10.1016/j.cell.2009.05.021

[26] Linkermann, A. et al. Dichotomy between RIP1- and RIP3-mediated necroptosis in tumor necrosis factor-alpha-induced shock. *Mol. Med.* **18**, 577–586 (2012). 22371307 10.2119/molmed.2011.00423 PMC3388137

[27] Ma, X. et al. The oncogenic microRNA miR-21 promotes regulated necrosis in mice. *Nat. Commun.* **6**, 7151 (2015). 25990308 10.1038/ncomms8151 PMC4440243

[28] Sendler, M., Mayerle, J. & Lerch, M. M. Necrosis, apoptosis, necroptosis, pyroptosis: it matters how acinar cells die during pancreatitis. *Cell. Mol. Gastroenterol. Hepatol.* **2**, 407–408 (2016). 28174728 10.1016/j.jcmgh.2016.05.007 PMC5042603

[29] Louhimo, J., Steer, M. L. & Perides, G. Necroptosis is an important severity determinant and potential therapeutic target in experimental severe pancreatitis. *Cell. Mol. Gastroenterol. Hepatol.* **2**, 519–535 (2016). 27642624 10.1016/j.jcmgh.2016.04.002 PMC5020563

[30] Ji, L. et al. Hydrogen sulphide exacerbates acute pancreatitis by over-activating autophagy via AMPK/mTOR pathway. *J. Cell. Mol. Med.* **20**, 2349–2361 (2016). 27419805 10.1111/jcmm.12928 PMC5134374

[31] Bai, X. et al. The apoptosis of peripheral blood lymphocytes promoted by hyperbaric oxygen treatment contributes to attenuate the severity of early stage acute pancreatitis in rats. *Apoptosis* **19**, 58–75 (2014). 24101212 10.1007/s10495-013-0911-x

[32] Lv, J. C., Wang, G., Pan, S. H., Bai, X. W. & Sun, B. Lycopene protects pancreatic acinar cells against severe acute pancreatitis by abating the oxidative stress through JNK pathway. *Free Radic. Res.* **49**, 151–163 (2015). 25410533 10.3109/10715762.2014.988150

[33] Wang, G., Sun, B., Gao, Y., Meng, Q. H. & Jiang, H. C. The effect of emodin-assisted early enteral nutrition on severe acute pancreatitis and secondary hepatic injury. *Mediat. Inflamm.* **2007**, 29638 (2007). 10.1155/2007/29638 PMC2220032 18288270

[34] Wang, G. et al. Protective effects of emodin combined with danshensu on experimental severe acute pancreatitis. *Inflamm. Res.* **59**, 479–488 (2010). 20043232 10.1007/s00011-009-0152-1

[35] Dlugosz, J. W., Andrzejewska, A., Nowak, K., Wroblewski, E. & Dabrowski, A. The cumulative effect of nuclear factor-kappaB (NF-kappaB) inhibition and endothelins in early cerulein-induced acute pancreatitis in rats. *Rocz. Akad. Med. Bialymst.* **50**, 230–236 (2005). 16358973

[36] Kan, S., Zhou, H., Jin, C. & Yang, H. Effects of PDTC on NF-kappaB expression and apoptosis in rats with severe acute pancreatitis-associated lung injury. *Int. J. Clin. Exp. Med.* **8**, 3258–3270 (2015). 26064215 PMC4443049

[37] Kusske, A. M., Rongione, A. J., Ashley, S. W., McFadden, D. W. & Reber, H. A. Interleukin-10 prevents death in lethal necrotizing pancreatitis in mice. *Surgery* **120**, 284–288 (1996). discussion 289. 8751594 10.1016/s0039-6060(96)80299-6

[38] Li, L. et al. Long Noncoding RNA MALAT1 Promotes Aggressive Pancreatic Cancer Proliferation and Metastasis via the Stimulation of Autophagy. *Mol. Cancer Ther.* **15**, 2232–2243 (2016). 27371730 10.1158/1535-7163.MCT-16-0008

[39] Kong, R. et al. Downregulation of nuclear factor-kappaB p65 subunit by small interfering RNA synergizes with gemcitabine to inhibit the growth of pancreatic cancer. *Cancer Lett*. **291**, 90–98 (2010). 19880242 10.1016/j.canlet.2009.10.001

[40] Bhatia, M. et al. Pathophysiology of acute pancreatitis. *Pancreatology* **5**, 132–144 (2005). 15849484 10.1159/000085265

[41] Kaiser, A. M. et al. Effects of cycloheximide on pancreatic endonuclease activity, apoptosis, and severity of acute pancreatitis. *Am. J. Physiol.* **271**, C982–C993 (1996). 8843729 10.1152/ajpcell.1996.271.3.C982

[42] Gukovskaya, A. S. et al. Cell death in pancreatitis: effects of alcohol. *J. Gastroenterol. Hepatol.* **21**(Suppl 3), S10–S13 (2006). 16958657 10.1111/j.1440-1746.2006.04571.x

[43] Bae, J. H., Shim, J. H. & Cho, Y. S. Chemical regulation of signaling pathways to programmed necrosis. *Arch. Pharm. Res.* **37**, 689–697 (2014). 24715577 10.1007/s12272-014-0385-6

[44] Wu, W., Liu, P. & Li, J. Necroptosis: an emerging form of programmed cell death. *Crit. Rev. Oncol. Hematol.* **82**, 249–258 (2012). 21962882 10.1016/j.critrevonc.2011.08.004

[45] Han, B. et al. MAPKs and Hsc70 are critical to the protective effect of molecular hydrogen during the early phase of acute pancreatitis. *FEBS J.* **283**, 738–756 (2016). 26683671 10.1111/febs.13629

[46] Newton, K. RIPK1 and RIPK3: critical regulators of inflammation and cell death. *Trends Cell Biol.* **25**, 347–353 (2015). 25662614 10.1016/j.tcb.2015.01.001

[47] Knepper, M. A., Kwon, T. H. & Nielsen, S. Molecular physiology of water balance. *N. Engl. J. Med.* **372**, 1349–1358 (2015). 25830425 10.1056/NEJMra1404726 PMC6444926

[48] Yang, M., Gao, F., Liu, H., Yu, W. H. & Sun, S. Q. Temporal changes in expression of aquaporin-3, -4, -5 and -8 in rat brains after permanent focal cerebral ischemia. *Brain Res.* **1290**, 121–132 (2009). 19616516 10.1016/j.brainres.2009.07.018

[49] Rabolli, V. et al. Critical role of aquaporins in interleukin 1beta (IL-1beta)-induced inflammation. *J. Biol. Chem.* **289**, 13937–13947 (2014). 24700466 10.1074/jbc.M113.534594 PMC4022865

[50] Wang, F., Huang, H., Lu, F. & Chen, Y. Acute lung injury and change in expression of aquaporins 1 and 5 in a rat model of acute pancreatitis. *Hepatogastroenterology* **57**, 1553–1562 (2010). 21443120

[51] Ko, S. B. et al. Aquaporin 1 water channel is overexpressed in the plasma membranes of pancreatic ducts in patients with autoimmune pancreatitis. *J. Med. Invest.* **56**(Suppl), 318–321 (2009). 20224214 10.2152/jmi.56.318

[52] Matsuzaki, T. et al. Aquaporins in the digestive system. *Med. Electron Microsc.* **37**, 71–80 (2004). 15221647 10.1007/s00795-004-0246-3

[53] Ma, T. & Verkman, A. S. Aquaporin water channels in gastrointestinal physiology. *J. Physiol.* **517**(Pt 2), 317–326 (1999). 10332084 10.1111/j.1469-7793.1999.0317t.x PMC2269340

[54] Ding, Z., Zhang, J., Xu, J., Sheng, G. & Huang, G. Propofol administration modulates AQP-4 expression and brain edema after traumatic brain injury. *Cell Biochem. Biophys.* **67**, 615–622 (2013). 23494261 10.1007/s12013-013-9549-0

