# Author Correction: Inhibition of RIPK1-dependent regulated acinar cell necrosis provides protection against acute pancreatitis via the RIPK1/NF-κB/AQP8 pathway

**DOI:** 10.1038/s12276-024-01171-9

**Published:** 2024-02-13

**Authors:** Peng-yu Duan, Yuan Ma, Xi-na Li, Feng-zhi Qu, Liang Ji, Xiao-yu Guo, Wang-jun Zhang, Fan Xiao, Le Li, Ji-sheng Hu, Bei Sun, Gang Wang

**Affiliations:** 1 Department of Pancreatic and Biliary Surgery, The First Affiliated Hospital of Harbin Medical University, Harbin, Heilongjiang Province China; 2 Department of Medical Administration, The First Affiliated Hospital of Harbin Medical University, Harbin, Heilongjiang Province China; 3 Department of Pharmacy, The First Affiliated Hospital of Harbin Medical University, Harbin, Heilongjiang Province China

Correction to: *Experimental & Molecular Medicine* 10.1038/s12276-019-0278-3, published online 02 August 2019

After online publication of this article, the authors noticed an error in the Fig. 7A and Fig. 7B section.

As fluorescence photography was taken on different working days, different sections were confused, leading to the problem of repeated field in Fig. 7. Since the fluorescent slides to be tested were all stored in the same box, when immunofluorescence indicators caspase-8 and caspase-9 were taken, the markers ‘AP’ and ‘AP+si-NC’ on the slide were confused, resulting in partial overlap in Fig. 7A. When TUNEL’s results were taken, the markers ‘AP+si-AQP8’ and ‘AP+si-NC’ on the slide were confused, resulting in partial overlap in Fig. 7B.

The incorrect figure is shown below.
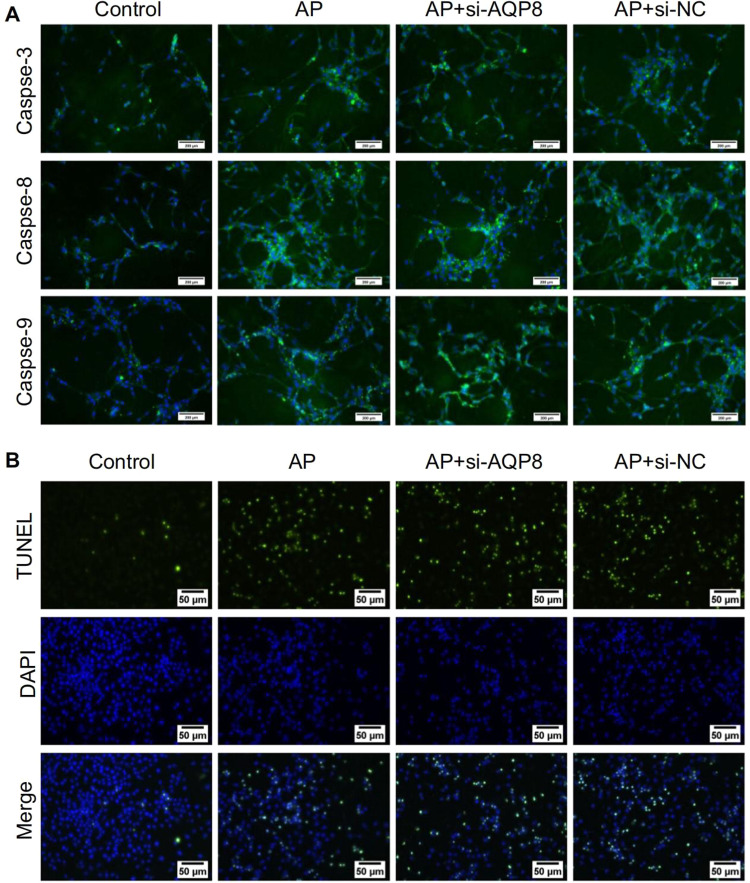


The correct figure is shown below.
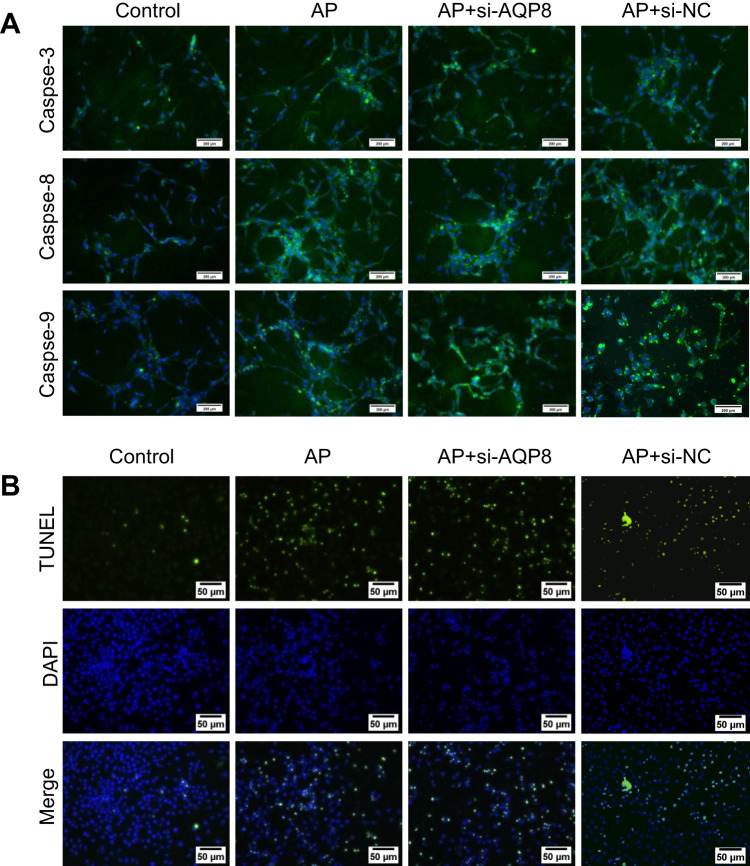


All the authors believe that the corrections would not affect any results, discussions and conclusions displayed in the rest of the article.

The authors apologize for any inconvenience caused.

The original article has been corrected.

