# Author Correction: Inhibition of RIPK1-dependent regulated acinar cell necrosis provides protection against acute pancreatitis via the RIPK1/NF-κB/AQP8 pathway

**DOI:** 10.1038/s12276-025-01471-8

**Published:** 2025-06-09

**Authors:** Peng-yu Duan, Yuan Ma, Xi-na Li, Feng-zhi Qu, Liang Ji, Xiao-yu Guo, Wang-jun Zhang, Fan Xiao, Le Li, Ji-sheng Hu, Bei Sun, Gang Wang

**Affiliations:** 1 Department of Pancreatic and Biliary Surgery, The First Affiliated Hospital of Harbin Medical University, Harbin, Heilongjiang Province China; 2 Department of Medical Administration, The First Affiliated Hospital of Harbin Medical University, Harbin, Heilongjiang Province China; 3 Department of Pharmacy, The First Affiliated Hospital of Harbin Medical University, Harbin, Heilongjiang Province China

Correction to: *Experimental & Molecular Medicine* 10.1038/s12276-019-0278-3, published online 2 August 2019

After online publication of this article, the authors noticed an error in the Fig.2C section.

Upon recent review, we identified another unintentional error in the transmission electron microscopy (TEM) images presented in Fig. 2C. Similar to the previous issue in Fig. 7, this mistake was caused by confusion between TEM sections obtained on different days, leading to duplication of images. Specifically, mislabeling and disorganized storage of image folders for the 'AP' and 'AP+DMSO' groups resulted in incorrect selection and unintended repetition during figure preparation.

We have updated the manuscript with the correct TEM images for Fig. 2C. All the authors believe that the correction would not affect any results, discussions and conclusions displayed in the rest of the article.
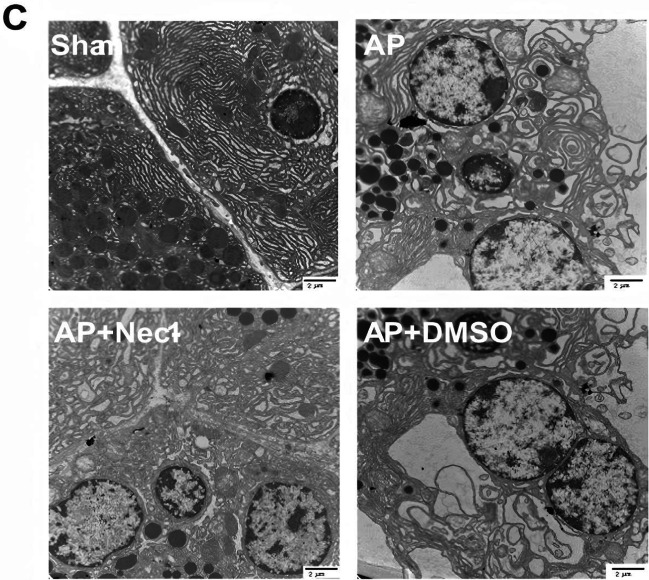


**Figure 2C.** Original.


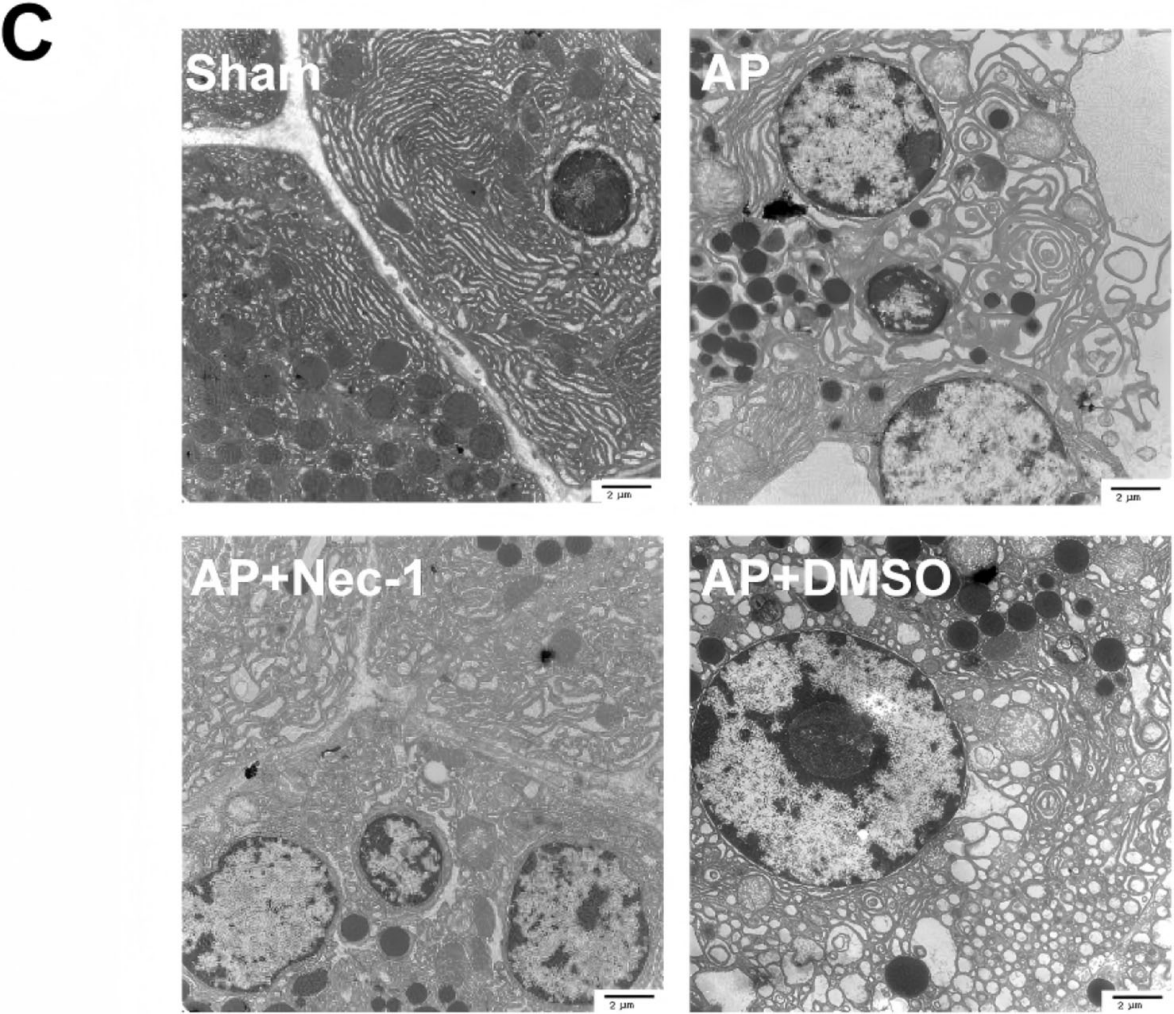


**Figure 2C.** Corrected.

The authors apologize for any inconvenience caused.

The original article has been corrected.

